# Interactions of Methylenetetrahydrofolate Reductase C677T Polymorphism with Environmental Factors on Hypertension Susceptibility

**DOI:** 10.3390/ijerph13060601

**Published:** 2016-06-17

**Authors:** Shujun Fan, Boyi Yang, Xueyuan Zhi, Yanxun Wang, Jian Wei, Quanmei Zheng, Guifan Sun

**Affiliations:** 1 Environment and Non-Communicable Disease Research Center, School of Public Health, China Medical University, Shenyang 110013, China; fanfan0721ykl@163.com (S.F.); zhixy90smile@126.com (X.Z.); qmzheng@mail.cmu.edu.cn (Q.Z.); 2 Guangzhou Key Laboratory of Environmental Pollution and Health Risk Assessment, Department of Preventive Medicine, School of Public Health, Sun Yat-sen University, Guangzhou 510080, China; yangby23@mail.sysu.edu.cn; 3 Division of Molecular Preventive Medicine, Shanghai Institute of Targeted Therapy and Molecular Medicine, Shanghai 200433, China; wangyanxun@genechina.com; 4 Brain Disease Center, Tianjin Dagang Oil Field General Hospital, Tianjin 300280, China; weijiantianjin@163.com

**Keywords:** environmental factors, hypertension, interaction, *MTHFR* C677T

## Abstract

Hypertension is considered to be the result of genes, environment, and their interactions. Among them age, sex, tobacco use, alcohol consumption, and being overweight/obesity are well documented environmental determinants, and methylenetetrahydrofolate reductase (*MTHFR*) C677T polymorphism is nominated as a potential genetic candidate. However, the synergistic effect of the *MTHFR* C677T polymorphism with these environmental factors on the risk of hypertension has received little attention. The aim of this study was to explore the associations of the *MTHFR* C677T polymorphism, environmental factors, and their interactions with hypertension predisposition in a Northern Chinese Han population. A total of 708 participants were enrolled in the study. The genotypes of the *MTHFR* C677T were determined by a TaqMan assay. We found that participants of an older age, being overweight/obesity, with a smoking habit, drinking habit, or carrying the 677T allele were at an increased risk of hypertension. Additionally, there existed marginally significant interactions of the polymorphism with age and overweight/obesity. However, future large, well-designed studies in Chinese and other populations, as well as mechanistic studies, are still needed to validate our findings, especially considering that the interactions observed in our study were only marginally significant.

## 1. Introduction

Hypertension, the leading risk factor for cardiovascular diseases and premature death, has become a severe public health challenge worldwide. In China, the estimated prevalence of hypertension among adults has increased from 7.5% in 1979 to 25.2% in 2012 [1]. However, the exact pathophysiology of hypertension remains unclear, and multiple factors are thought to contribute to the development and progression of the disorder. Established environmental determinants of blood pressure levels include age, sex, tobacco use, sodium intake, alcohol consumption, dietary habits, being overweight/obesity, and dyslipidemia [2,3,4]. In addition to environmental factors, genetic factors also have been identified as modulators of hypertension. Hundreds of genes and polymorphisms have been hypothesized to be involved in the pathogenesis of hypertension, and it is estimated that 30%–40% of the variation in blood pressure could be attributed to heritability [5]. A recent genome-wide association study has linked genetic variation at eight loci with high blood pressure, including the gene encoding methylenetetrahydrofolate reductase (*MTHFR*), an enzyme catalyzing the reduction of 5,10-methylenetetrahydrofolate to 5-methyltetrahydrofolate [6]. The enzyme resides at an important metabolic branch point directing the distribution of folate derivatives to meet requirements for homocysteine and DNA methylation or for DNA and RNA biosynthesis. C677T is a common polymorphism in the *MTHFR* gene. The incidence of this mutation in the homozygous and heterozygous state is 43.9% and 23.2% in the Chinese population, respectively, which is higher compared with many other populations worldwide [7]. As a result of this polymorphism, the 677TT carriers have 30% and 677CT carriers have 60% of the wild-type (677CC) enzymatic activity, causing impaired remethylation of homocysteine to methionine and subsequent hyperhomocysteinemia, especially under conditions of low dietary folate [8]. Hyperhomocysteinemia has been linked to hypertension as it may induce arteriolar constriction, renal dysfunction, increased sodium reabsorption, and increase arterial stiffness and oxidative stress [9]. The 677T allele could also cause DNA hypomethylation [10], which has been involved in the pathogenesis of hypertension [11]. Therefore, the *MTHFR* C677T polymorphism is expected to be potentially associated with hypertension.

Previously, numerous epidemiological studies have explored the relationship of the *MTHFR* C677T polymorphism with hypertension, but the results were inconsistent [12]. Our previous large meta-analysis including 114 studies found that the polymorphism was significantly related to hypertension among East Asians and Caucasians, but not among Latinos, Black Africans, Indians, and Sri Lankans [12]. Several genome-wide association studies, mostly among Europeans, have identified more than 30 genomic loci linked to blood pressure levels [6,13]; however, subsequent genome-wide association studies among East Asians showed inconsistent results [14,15]. Cumulative effect of the genetic loci identified through these genome-wide association studies explained <2.5% of systolic and diastolic blood pressure variance despite estimates of heritability of blood pressure of 30%–40% [5,13,16]. These inconsistent observations may be explained by a number of hypotheses, with gene-environment interaction emerging as a strong candidate because genetic effects on blood pressure can be altered by environmental exposures via multiple biological pathways.

In recent years, greater emphasis has been placed on the joint effects of genetic and environmental factors on complex disease traits, including hypertension. Two recent studies respectively investigated gene-smoking and gene-alcohol interaction effects on blood pressure using genome-wide data from the Framingham Heart Study and identified several novel blood pressure loci [4,16]. Xi *et al.* investigated the effect of obesity on relationship of six genetic loci recently identified by genome-wide association studies with hypertension among Chinese children and found five loci were significantly associated with hypertension only in obese individuals [17]. A study in Brazil revealed that angiotensinogen Met235Thr and endothelial nitric oxide synthase Glu298Asp polymorphisms had interactions with smoking, sedentary lifestyle, elevated total cholesterol, and older age towards the development of hypertension [18]. For the *MTHFR* C677T polymorphism, its interaction with B-vitamins on cardiovascular diseases, hypertension, and blood pressure lowering has also been extensively investigated [19,20,21]. For example, Klerk *et al.* found that the 677TT genotype was associated with an increased risk of coronary heart disease in Europeans but not in North Americans, which was likely to be driven by differences in exposure to B-vitamins, especially folate intake [19]. In addition, Wilson and colleagues demonstrated that riboflavin supplementation is effective in reducing blood pressure specifically in individuals with the 677TT genotype [21]. However, the influence of other potential risk factors (such as age, being overweight/obesity, alcohol drinking, and cigarette smoking) on association between the *MTHFR* C677T polymorphism and the risk of hypertension received little attention, especially considering that these factors have been shown to, independently or in combination with the *MTHFR* C677T polymorphism, affect homocysteine levels [22]. Hence, we performed a cross-sectional study to explore the associations of some environmental factors, the *MTHFR* C677T polymorphism, and their interactions with hypertension predisposition in a Northern Chinese Han population.

## 2. Materials and Methods

### 2.1. Study Population and Inclusion Criteria

The study was carried out in the physical examination center of the Dagang Oil Field General Hospital, Tianjin Municipality. We used random number tables (by using SAS version 9.2, SAS Institute Inc., Cary, NC, USA) to select study subjects from persons who took regular health examinations during the period between 2010 and 2012. Inclusion criteria were as follows: (1) age ≥18 years; (2) Han nationality; (3) without severe chronic or infectious diseases. In the beginning, 835 persons expressed an interest in our study and agreed to undergo a genotype determination. Then, based on the above inclusion criteria, 127 subjects were excluded, of which 75 had incomplete data and 52 reported histories of diabetes mellitus, cardiovascular diseases, renal diseases, myocardial infarction, or secondary hypertension. Finally, 708 participants were included in formal analysis (Table 1). The participants included both households and working populations in the Dagang Oil Field. The study was conducted in accordance with the Helsinki Declaration, and the protocols were approved by the ethics review committee of the China Medical University (Shenyang, China; Identification code: CMU 62073024; 15 July 2008). Informed consent was obtained from all participants prior to study entry.

### 2.2. Data Collection and Definitions

We used a self-administered questionnaire to collect data on demographic characteristics, smoking and drinking status, and other health-related information. Smoking status was categorized as current smokers (smoking during the last one year or quit smoking less than six months ago), former smokers (quit smoking more than six months ago), or non-smokers (never smoke). Drinking status were categorized as current drinkers (at least two times/week for men and one time/week for women), former drinkers (quit drinking more than six months ago), or non-drinkers (never drink).

Body weight, height, and waist circumference were measured using a standard scale with light clothing and barefoot. Body mass index (BMI) was calculated as weight in kilograms divided by the square of height in meters (kg/m^2^). Overweight/obesity was defined as a BMI ≥ 24 according to the Guidelines on the Prevention and Management of Overweight and Obesity in Adults: China [23]. Blood pressure was measured according to standardized procedural guidelines [24] while subjects were in the sitting position after 15 min of rest using standardized mercuric-column sphygmomanometer with appropriate adult cuff size by a carefully trained nurse. Systolic blood pressure (SBP) and diastolic blood pressure (DBP) were determined by the first and the fifth Korotkoff sounds. The average of three consecutive measurements to the nearest 2 mmHg was recorded, with a time interval of at least two minutes. All participants were requested not to consume tea, coffee, alcohol, or tobacco, and to exercise for at least 30 min before measuring their blood pressure. Essential hypertension was regarded as the mean SBP at least 140 mmHg and/or DBP at least 90 mmHg and/or currently receiving treatment for hypertension. Normotension was regarded as the mean SBP/DBP at most 120/80 mmHg and with no history of hypertension.

The concentrations of triglyceride (TG), cholesterol (TC), high density lipoprotein (HDL), low density lipoprotein (LDL), and fasting blood glucose (FBG) were determined by enzymatic method using a Hitachi Autoanalyzer (Type 7170A; Hitachi Ltd., Tokyo, Japan) [25].

### 2.3. Genotyping

Genomic DNA was extracted from buccal samples using QiAamp DNA Mini Kit (Qiagen, Valencia, CA, USA). The genotypes of the *MTHFR* C677T were determined by a TaqMan assay, which has been detailed in our previous paper [7].

### 2.4. Statistical Analysis

Differences in the distribution of baseline characteristics between hypertensive and normotensive groups were tested using student’s t-test for continuous variables (SBP, DBP, TC, TG, HDL, LDL, and FBG) and chi-square test for categorical variables (age, gender, smoking status, drinking status, and BMI). All of the continuous variables were tested for normality before Student’s t-test, and those non-normally distributed variables were log-transformed to reach normality and variance homogeneity. Logistic regression analysis was used to examine the associations of age, gender, BMI, drinking, and smoking status with hypertension susceptibility. We coded age ≤45 years = 0, and age >45 years = 1 (the mean age of the study subjects was 45.60 years); normal weight = 0, and overweight/obesity = 1. As the number of former-drinkers and former-smokers were too small to deduce valid results, we coded non-drinkers = 0, ever-drinkers (current-drinkers and former-drinkers) = 1, and non-smokers = 0, ever-smokers (current-smokers and former-smokers) = 1. A goodness of fit chi-square test was used to evaluate whether the genotypic distribution of the *MTHFR* C677T polymorphism was in accordance with Hardy-Weinberg equilibrium. We also used logistic regression analysis to examine the potential effects of the *MTHFR* C677T polymorphism on hypertension risk under five genetic models (homozygous codominant, heterozygous codominant, dominant, recessive, and allelic models). Then, we used the methods suggested by Thakkinstian *et al.* to select the most appropriate genetic model [26].

Subsequently, multivariable-adjusted stratified analyses were used to explore potential gene-environmental interactions based on the most appropriate genetic model. The *MTHFR* C677T polymorphism was dichotomized under dominant genetic model (the selected genetic model) in which TT and CT genotypes were combined, and we use the combined genotype as the “at risk” genotype for easy interpretation. Similarly, environmental characteristics were dichotomized by appropriate grouping noted above. Furthermore, relative excess risk due to interaction (RERI) and corresponding 95% confidence interval (CI) were calculated to evaluate the presence of interactions on the additive scale. The RERI is a measure of difference in excessive relative risks, and we calculated RERI and its 95% CI using an Excel sheet downloaded from Epidemiological net [27]. If absent an interaction effect, RERI = 0; if presenting a positive interaction, RERI > 0; and if presenting a negative interaction, RERI < 0. Linear regression analysis was also used to examine the association between age and blood pressure in each *MTHFR* C677T genotype group. In addition to the calculations of RERI and its 95% CI, all other statistical analyses were conducted using SAS software (version 9.2, SAS Institute Inc., Cary, NC, USA). A two tailed *p* value less than 0.05 was taken as statistically significant.

## 3. Results

### 3.1. General Characteristics of the Study Population

A total of 708 subjects including 518 males and 190 females were finally included in the present study. Table 1 summarizes the baseline characteristics of these participants. The mean age of the study population was 45.60 ± 11.03 years. Compared with the normotensive controls, hypertensive individuals had significantly higher SBP, DBP, TG, TC, LDL, and FBG levels, and they were more likely to be older, men, overweight/obese, former smokers, and ever-drinkers (all *p* < 0.05).

### 3.2. Association between Environmental Factors and Hypertension Susceptibility

Table 2 shows the results of univariate and multivariate logistic regression analyses for hypertension in relation to environmental factors including age, gender, BMI, and smoking and drinking status. In the univariate model, age >45 years, being male, overweight/obesity, ever-smokers, and ever-drinkers were significantly associated with an increased risk of hypertension (*p* = 0.038 to <0.001). After adjustment for multiple covariates, the association of age >45 years, being male, overweight/obesity, and ever-drinkers with hypertension was still statistically significant with an OR of 2.63 (95% (CI): 1.84–3.76, *p* < 0.001), 1.81 (95% CI: 1.08–3.04, *p* = 0.025), 2.78 (95% CI: 1.83–4.22, *p* < 0.001), and 1.60 (95% CI: 1.10–2.33, *p* = 0.015), respectively. However, the relationship of smoking status with hypertension failed to reach significance (*p* = 0.743).

### 3.3. Association between MTHFR C677T Polymorphism and Hypertension Susceptibility

Genotypic and allelic frequencies for the *MTHFR* C677T polymorphism are summarized in Table 3. The genotypic distribution of the polymorphism among the study population was in accordance with the Hardy-Weinberg equilibrium (*p* = 0.20). The frequency of the *MTHFR* 677T allele in the hypertensive group (60.77%) was significantly higher than the normotensive group (53.76%) (*p* = 0.004). We further investigated the associations of the *MTHFR* C677T polymorphism with hypertension risk using the unconditional logistic regression analysis under five genetic models. In the univariate analysis, the *MTHFR* C677T polymorphism was significantly associated with hypertension under the homozygous co-dominant, dominant, and allelic models. After adjustments for age, gender, BMI, and smoking and drinking status, significant association was still observed in homozygous co-dominant (OR = 1.81, 95% CI = 1.10–2.98, *p* = 0.020) and dominant (OR = 1.64, 95% CI = 1.05–2.55, *p* = 0.029) models.

### 3.4. Interaction Effects of the MTHFR C677T Polymorphism with Environmental Factors on Hypertension Susceptibility

Table 4 shows the joint effects of the *MTHFR* C677T polymorphism with dichotomous age, gender, BMI, smoking status, and drinking status on hypertension risk. Compared with younger subjects with CC genotype, the hypertension risk among subjects who were older and carried the “at risk” genotype (OR = 4.08, 95% CI = 1.95–8.54, *p* < 0.001) was much greater than those who were older with CC genotype (OR = 2.36, 95% CI = 1.02–5.47) and among subjects who were younger with the “at risk” genotype (OR = 1.46, 95% CI = 0.69–3.12). The corresponding RERI was 1.26 (95% CI = −0.33–2.85, *p* = 0.103), suggesting a marginally significant interaction on the additive level.

**Figure 1:**
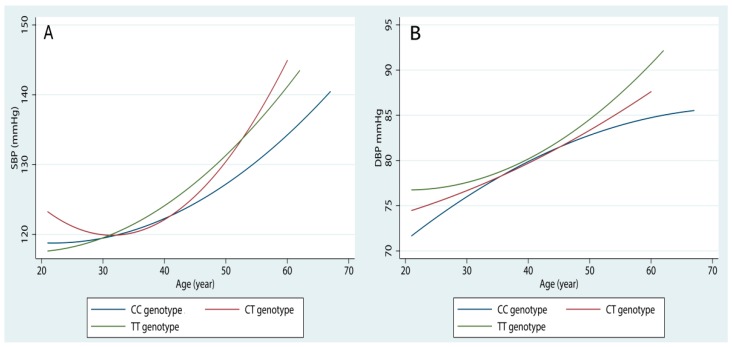
The effects of age on SBP (**A**) and DBP (**B**) levels in different *MTHFR* C677T genotype groups.

In addition, we found that the strongest effects of age on blood pressure levels was in the 677TT genotype carriers, followed by 677CT genotype and 677CC genotype carriers (Figure 1). Similarly, there were some indications for an interaction between the polymorphism and being overweight/obesity. The joint OR for overweight/obesity and the “at risk” genotype was 3.90 (95% CI = 1.74–8.72, *p* = 0.001), which is greater than the independent ORs for the “at risk” genotype (OR = 1.34, 95% CI = 0.56–3.19) and overweight/obesity (OR = 2.23, 95% CI = 0.91–5.48), with an RERI of 1.33 (95% CI = −0.15–2.80, *p* = 0.084). No apparent combined effect between sex, drinking status, smoking status, and the polymorphism was found.

## 4. Discussion

In this study we systematically explored the effects of the *MTHFR* C677T polymorphism, several environmental factors, and their interactions on the development of hypertension among the Northern Chinese Han population. Participants with older age, high BMI, smoking habits, drinking habits, or carrying the 677T allele were at increased risk of hypertension. Furthermore, we observed some indications for interactions of the *MTHFR* C677T mutant genotype with older age and being overweight/obesity.

Numerous prior epidemiological studies have investigated the association of the *MTHFR* C677T polymorphism with hypertension, but the results were controversial [12,28]. A recent meta-analysis by Niu *et al.* included nine studies conducted among the Chinese population, four of which reported a significant association between the C677T polymorphism and essential hypertension, but the remaining five did not [28]. Our research group also demonstrated differences in hypertension risk in relation to the polymorphism among different populations worldwide [12]. In the present study, we observed that the 677T allele was significantly associated with an increased risk of hypertension in the Northern Chinese Han population. Many factors may contribute to the phenomenon that the *MTHFR* C677T polymorphism is related to hypertension in one population, but not in another population. Among them, gene-environment interaction could not be neglected as our current understanding of pathogenesis of hypertension indicates a multifactorial and multistep process including various genetic and environmental factors.

We further explored possible interaction effects of the *MTHFR* C677T polymorphism with several environmental factors, and found that the joint effects of the “at risk” genotype with older age and being overweight/obesity were greater than the sum of their individual effects. The RERIs for the interaction of older age and being overweight/obesity with the “at risk” genotype were 1.26 (95% CI = −0.33–2.85) and 1.33 (95% CI = −0.15–2.80), respectively, indicating an OR that is 1.26 and 1.33 times higher, respectively, as a result of the interaction. Notably, the calculated lower confidence limits for both RERIs were less than, but close, to zero, meaning that the interactions were marginally significant. One possible explanation for the marginally significant interaction might be the relatively small sample size of the present study, especially in some subgroups.

The precise mechanisms by which age and the *MTHFR* C677T polymorphism interact to influence the development of hypertension remain unclear. However, both older age and the 677T allele can cause hyperhomocysteinemia and aberrant DNA methylation [10,29,30,31], which are significantly associated with hypertension [9,11]. Furthermore, an interaction between age and the *MTHFR* C677T polymorphism on plasma homocysteine concentrations has been reported [32]. Therefore, the synergistic effect of the *MTHFR* C677T polymorphism and age on hypertension may be partially due to their interactive effects on homocysteine concentrations and DNA status. The age-gene synergism observed in this study may be biologically plausible and expected.

Consistent with our findings on the potential interaction between the polymorphism and being overweight/obesity, a large population-based case-control study among Chinese population by Xi *et al.* observed that the *MTHFR* C677T polymorphism was significantly associated with hypertension only in obese children [17]. Overweight/obesity can increase the risk of hypertension by activating the sympathetic nervous system and renin-angiotensin system, inducing insulin resistance, and impairing endothelial function [33]. Some of these pathophysiological mechanisms such as insulin resistance and endothelial dysfunction are shared by hyperhomocysteinemia in causing hypertension [9]. Additionally, several epidemiological studies observed that overweight/obesity subjects had higher homocysteine and/or lower folate levels than normal weight ones [34,35]. Furthermore, a body of evidence has suggested that elevated homocysteine levels might cause the development of being overweight/obesity via epigenetic control gene expression in the regulation of body fat storage [36,37]. Furthermore, a recent study by Yin *et al.* showed that the *MTHFR* C677T polymorphism interacted with overweight/obesity to modulate serum lipid levels [38]. In the present study, we also evaluated possible joint effects of the polymorphism and being overweight/obesity on serum lipid levels and found that subjects carrying the “at risk” genotype and being overweight/obesity have significantly higher TC and TG levels and lower HDL levels than other subjects (data not shown). Dyslipidemia has a causal relationship with hypertension. So we inferred that the interaction of the *MTHFR* C677T polymorphism and overweight/obesity on dyslipidemia may be a partial reason for their joint effects on hypertension found in this study. In spite of this, the biological mechanisms by which overweight/obesity modifies the relationship between the *MTHFR* C677T polymorphism and hypertension risk remain unclear and just can be speculated. Further studies are therefore still warranted to investigate the precise mechanism of the interaction in the pathogenesis of hypertension.

The relationship between cigarette smoking and hypertension is controversial. Some investigators reported that cigarette smoking can increase the risk of hypertension, while others reported that it was associated with reduced hypertension risk, and some found no direct association between them [39,40,41]. However, cigarette smoking was demonstrated to play a role in modifying the effects of some genes on hypertension [42]. For example, Yin *et al.* observed no association between cigarette smoking and hypertension in a Guangxi Bai Ku Yao population; however, they found that cigarette smoking showed interaction with the *MTHFR* C677T polymorphism to affect blood pressure levels [42]. In contrast, our results showed no interaction between cigarette smoking and the polymorphism. Alcohol consumption (especially excessive alcohol consumption) is a known risk factor for hypertension, which was also confirmed in our study. The intermediate metabolites of alcohol can change genes directly and modulate their expression via epigenetic mechanisms [43]. Therefore, interaction between alcohol drinking and genes may be biologically plausible. Another analysis by Yin *et al.* found interactive effects of alcohol consumption with the *MTHFR* C677T polymorphism on hypertension [43], which was different from our findings. The discrepancies between our findings and those reported by Yin *et al.* [42,43] can be caused by many factors, including differences in ethnic background (Bai Ku Yao [42,43] *vs.* Han (the present study)), sample size, dietary habits, and other environmental exposures.

In interpreting the findings of our study, several limitations need to be considered. Firstly, this study used a cross-sectional design, which precludes causal inference. Secondly, the study subjects were recruited from only one hospital, which compromises the representation of the general population and our findings may not be generalizable to the whole Chinese population, although the genotype distribution of the polymorphism in our study subjects complied with the Hardy-Weinberg equilibrium. Thirdly, selection bias is possible because the majority of the study participants were workers in the Dagang Oil Field, and there were significantly more male subjects than females. The significant gender difference in the number of study subjects also makes it difficult to draw conclusions regarding gender. Fourthly, because we used a questionnaire to collect personal exposure information (such as smoking and drinking), the responses might be influenced by the participant’s memory, so recall bias cannot be excluded. Fifthly, some information on relevant potential confounders, such as menopause status, dietary habits (especially folate and riboflavin intake), physical activity, and other genes were not included and controlled in this study. This could have affected the accuracy of our estimates. Despite these limitations, our study still has several apparent advantages. First, the study was carried out in a northern region where 677TT genotype frequency was high according to our previous findings [7], which guarantees sufficient number of 677TT genotype carriers to enhance the statistical power and to save the research costs. Second, all participants were of Han nationality, which reduces the potential effects of population stratification and improves the validity of statistical analysis. Third, to the best of our knowledge, this is the first study to explore and report the joint effects of the *MTHFR* C677T polymorphism with age, being overweight/obesity, gender, alcohol drinking, and cigarette smoking on hypertension predisposition.

## 5. Conclusions

In summary, the results of the present study suggest that the *MTHFR* 677T allele is associated with an increased risk of hypertension. Additionally, these results suggest a potential synergistic effect of the polymorphism with age and BMI on hypertension susceptibility. Our findings provide a further insight on the gene-environment relationships involved in hypertension pathogenesis. However, future large, well-designed studies in Chinese and other populations, as well as mechanistic studies, are needed to validate our findings, especially considering that the interactions observed in our study were marginally significant.

## Figures and Tables

**Table 1:** The characteristics of the study participants.

Characteristics	Total	Hypertensive	Normotensive	*p*-Value
Age (years), *n* (%)				<0.001
≤45	350 (49.44)	66 (30.84)	284 (57.49)	
>45	358 (50.56)	148 (69.16)	210 (42.51)	
Mean age (years)				
≤45	38.99 ± 5.54	39.70 ± 4.97	38.83 ± 5.66	0.251
>45	51.73 ± 4.44	52.34 ± 4.38	51.30 ± 4.44	0.027
Gender, *n* (%)				<0.001
Female	190 (26.84)	26 (12.15)	164 (33.20)	
Male	518 (73.16)	188 (87.85)	330 (66.80)	
Smoking Status, *n* (%)				0.017
Never smoker	448 (63.28)	121 (56.54)	327 (66.19)	
Former smoker	27 (3.81)	13 (6.07)	14 (2.83)	
Current smoker	233 (32.91)	80 (37.38)	153 (30.97)	
Drinking Status, *n* (%)				0.038
Never drinker	443 (62.57)	106 (49.53)	337 (68.22)	
Former drinker	10 (1.41)	5 (2.34)	5 (1.01)	
Current drinker	255 (36.02)	103 (48.13)	152 (30.77)	
BMI (kg/m^2^), *n* (%)				<0.001
Normal	266 (37.57)	38 (17.76)	228 (46.15)	
Overweight/obesity	442 (62.43)	176 (82.24)	266 (53.85)	
SBP (mm Hg)	127.49 ± 18.70	148.38 ± 15.40	118.44 ± 11.30	<0.001
DBP (mm Hg)	82.00 ± 13.48	96.91 ± 9.93	75.53 ± 8.93	<0.001
TC (mmol/L)	5.14 ± 0.95	5.35 ± 0.96	5.05 ± 0.93	<0.001
TG (mmol/L) *	1.13 (0.75, 1.73)	1.39 (0.97, 2.11)	1.02 (0.70, 1.47)	<0.001
HDL-C (mmol/L)	1.34 ± 0.33	1.31 ± 0.33	1.35 ± 0.33	0.132
LDL-C (mmol/L)	2.97 ± 0.94	3.10 ± 0.99	2.92 ± 0.91	0.019
FBG (mmol/L)	5.29 ± 0.93	5.54 ± 1.07	5.19 ± 0.84	<0.001

Abbreviations: BMI, body mass index; SBP, systolic blood pressure; DBP, diastolic blood pressure; FBG, fasting blood glucose; TC, cholesterol; TG, triglycerides; HDL-C, high-density lipoprotein cholesterol; LDL-C, low-density lipoprotein cholesterol. * Values are expressed as median and range.

**Table 2:** Associations of age, gender, BMI, smoking, and drinking status with the risk of essential hypertension.

Characteristics	Hypertensive	Normotensive	Crude OR (95% CI)	*p*-Value	Adjusted OR (95% CI)	*p*-Value
Age						<0.001
≤45	66	284	1.0 (reference)		1.0 (reference)	
>45	148	210	3.03 (2.16–4.26)	<0.001	2.63 (1.84–3.76) ^a^	
Gender						0.025
Female	26	164	1.0 (reference)		1.0 (reference)	
Male	188	330	3.59 (2.29–5.64)	<0.001	1.81 (1.08–3.04) ^b^	
BMI						<0.001
Normal	38	228	1.0 (reference)		1.0 (reference)	
Overweight/Obesity	176	266	3.97 (2.68–5.88)	<0.001	2.78 (1.83–4.22) ^c^	
Smoking status						0.743
Non-smokers	119	327	1.0 (reference)		1.0 (reference)	
Ever-smokers	95	167	1.56 (1.13–2.17)	0.008	1.07 (0.73–1.56) ^d^	
Drinking status						0.015
Non-drinkers	105	337	1.0 (reference)		1.0 (reference)	
Ever-drinkers	109	157	2.23 (1.61–3.09)	<0.001	1.60 (1.10–2.33) ^e^	

Abbreviations: BMI, body mass index; OR, odds ratio; CI, confidence interval. Adjusted OR: ^a^ adjusted for gender, BMI and smoking, drinking status; ^b^ adjusted for age, BMI and smoking, drinking status; ^c^ adjusted for age, gender and smoking, drinking status; ^d^ adjusted for age, gender, BMI and drinking status; ^e^ adjusted for age, gender, BMI and smoking status. *p*-Value < 0.05 was considered statistically significant.

**Table 3:** Association of the *MTHFR* C677T polymorphism with risk of essential hypertension.

*MTHFR* C677T	Hypertension, *n* (%)	Normotensive, *n* (%)	Crude OR (95% CI)	*p*-Value	Adjusted OR (95% CI)	*p*-Value
Codominant						
CC	37 (17.29)	119 (24.09)	1.0 (reference)		1.0 (reference)	
CT	102 (47.66)	234 (47.37)	1.40 (0.91–2.17)	0.129	1.53 (0.96–2.45)	0.075
TT	75 (35.05)	141 (28.54)	1.71 (1.08–2.72)	0.023	1.81 (1.10–2.98)	0.020
Dominant						
CC	37 (17.29)	119 (24.09)	1.0 (reference)		1.0 (reference)	
CT + TT	177 (82.71)	375 (75.91)	1.52 (1.01–2.29)	0.046	1.64 (1.05–2.55)	0.029
Recessive						
CC + CT	139 (64.95)	353 (71.46)	1.0 (reference)		1.0 (reference)	
TT	75 (35.05)	141 (28.54)	1.35 (0.96–1.90)	0.085	1.34 (0.93–1.94)	0.120
Allele						
C	176 (41.12)	472 (47.77)	1.0 (reference)			
T	252 (58.88)	516 (52.23)	1.31 (1.04–1.65)	0.021		

Abbreviations: *MTHFR*, methylenetetrahydrofolate reductase; OR, odds ratio; CI, confidence interval. Adjusted OR: adjusted for age, gender, BMI, smoking and drinking status. *p*-Value < 0.05 was considered statistically significant.

**Table 4:** Interaction effects of the *MTHFR* C677T polymorphism with age, sex, BMI, drinking, and smoking status on hypertension susceptibility.

Genotype	Demographic and Lifestyle Factor	Adjusted OR * (95% CI)	*p*-Value	RERI (95% CI)	*p*-Value
Age					
CC	≤45	1.0 (reference)		1.26 (−0.33–2.85)	0.103
CT + TT		1.46 (0.69–3.12)	0.325		
CC	>45	2.36 (1.02–5.47)	0.045		
CT + TT		4.08 (1.95–8.54)	<0.001		
Sex					
CC	Female	1.0 (reference)		−0.27 (−2.85–2.32)	0.818
CT + TT		2.77 (0.77–9.99)	0.119		
CC	Male	2.98 (0.82–10.85)	0.098		
CT + TT		4.48 (1.29–15.55)	0.018		
BMI					
CC	Normal weight	1.0 (reference)		1.33 (−0.15–2.80)	0.084
CT + TT		1.34 (0.56–3.19)	0.506		
CC	Overweight/obesity	2.23 (0.91–5.48)	0.079		
CT + TT		3.90 (1.74–8.72)	0.001		
Drinking status					
CC	Non-drinkers	1.0 (reference)		0.40 (−0.92–1.72)	0.569
CT + TT		1.63 (0.89–2.97)	0.111		
CC	Ever-drinkers	1.59 (0.72–3.55)	0.255		
CT + TT		2.62 (1.39–4.96)	0.003		
Smoking status					
CC	Non-smokers	1.0 (reference)		−0.88 (−2.51–0.75)	0.271
CT + TT		2.07 (1.17–3.65)	0.012		
CC	Ever-smokers	1.70 (0.74–3.88)	0.211		
CT + TT		1.88 (1.04–3.42)	0.038		

Abbreviations: *MTHFR*, methylenetetrahydrofolate reductase; BMI, body mass index; OR, odds ratio; CI, confidence interval; RERI, relative excess risk due to interaction. * Joint ORs for age and genotype was adjusted by sex, BMI, drinking and smoking status; joint ORs for gender and genotype was adjusted by age, BMI, drinking and smoking status; joint ORs for BMI and genotype were adjusted by age, sex, drinking, and smoking status; joint ORs for drinking status and genotype was adjusted by age, sex, BMI and smoking status; joint ORs for smoking and genotype were adjusted by age, sex, and drinking status. *p*-Value < 0.05 was considered statistically significant.

## Abbreviations

The following abbreviations are used in this manuscript:

BMI	body mass index
CI	confidence index
DBP	diastolic blood pressure
FBG	fasting blood glucose
GWAS	genome-wide association study
HDL-C	high-density lipoprotein
LDL-C	low-density lipoprotein
*MTHFR*	methylenetetrahydrofolate reductase
OR	odds ratio
RERI	relative excess risk due to interaction
SBP	systolic blood pressure
TC	total cholesterol
TG	triglyceride
